# Supplementary material for: The causal role of circulating inflammatory markers in osteoporosis: a bidirectional Mendelian randomized study
Source: Front Immunol. 2024 Jul 18;15:1412298. doi: 10.3389/fimmu.2024.1412298 (PMC11291241; doi:10.3389/fimmu.2024.1412298)
Supplement: Supplementary file 1 [file DataSheet_1.zip › supplementary material/OSTEOPOROSIS-CIM/GCST90274835.tsv.gz_forest.pdf]

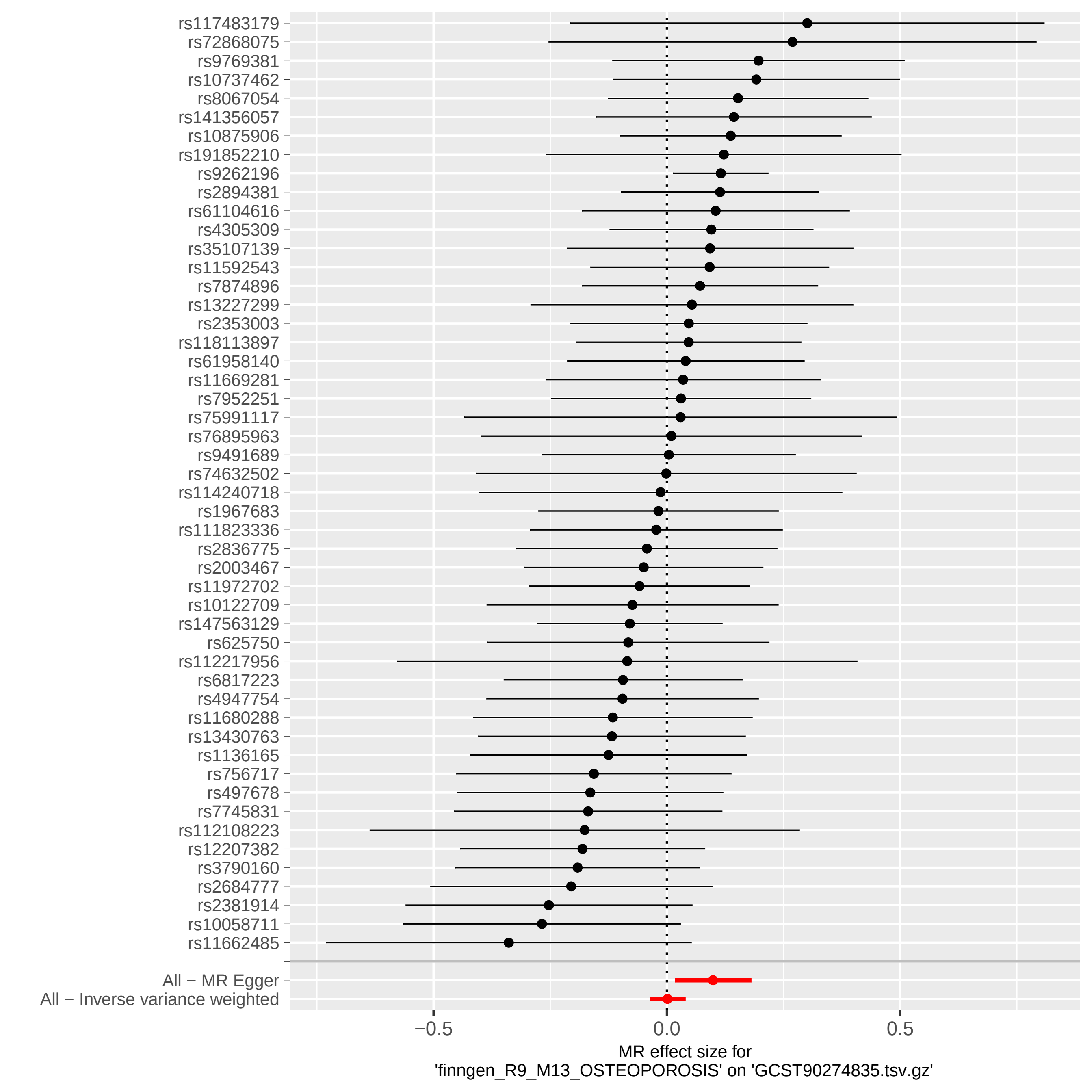

rs117483179
rs72868075
rs9769381
rs10737462
rs8067054
rs141356057
rs10875906
rs191852210
rs9262196
rs2894381
rs61104616
rs4305309
rs35107139
rs11592543
rs7874896
rs13227299
rs2353003
rs118113897
rs61958140
rs11669281
rs7952251
rs75991117
rs76895963
rs9491689
rs74632502
rs114240718
rs1967683
rs111823336
rs2836775
rs2003467
rs11972702
rs10122709
rs147563129
rs625750
rs112217956
rs6817223
rs4947754
rs11680288
rs13430763
rs1136165
rs756717
rs497678
rs7745831
rs112108223
rs12207382
rs3790160
rs2684777
rs2381914
rs10058711
rs11662485
All − MR Egger
All − Inverse variance weighted
−0.5
0.0
0.5
MR effect size for
'finngen_R9_M13_OSTEOPOROSIS' on 'GCST90274835.tsv.gz'
